# 2,2′-Bis{8-[(benzyl­amino)­methyl­idene]-1,6-dihy­droxy-5-isopropyl-3-methyl­naphthalen-7(8*H*)-one}

**DOI:** 10.1107/S1600536813027281

**Published:** 2013-10-09

**Authors:** Shukhrat M. Hakberdiev, Samat A. Talipov, Davranbek N. Dalimov, Bakhtiyar T. Ibragimov

**Affiliations:** a National University of Uzbekistan, Faculty of Chemistry, Vuzgorodok, Tashkent 100095, Uzbekistan; b Institute of Bioorganic Chemistry, Mirzo-Ulugbek St. 83, Tashkent 100125, Uzbekistan

## Abstract

The asymmetric unit of the title compound, C_44_H_44_N_2_O_6_, contains two independent mol­ecules with similar conformations. The di­hydro­naphthalene ring systems are approximately planar [maximum deviations = 0.036 (2), 0.128 (2), 0.0.24 (2) and 0.075 (2) Å]. The dihedral angle between two di­hydro­naphthalene ring systems is 83.37 (4)° in one mol­ecule and 88.99 (4)° in the other. The carbonyl O atom is linked with the adjacent hy­droxy and imino groups *via* intra­molecular O—H⋯O and N—H⋯O hydrogen bonds. In the crystal, mol­ecules are linked through O—H⋯O hydrogen bonds into layers parallel to (001), and adjacent layers are further stacked by π–π inter­actions between di­hydro­naphthalene and phenyl rings into a three-dimensional supra­molecular architecture. In the crystal, one of the isopropyl groups is disordered over two positions with an occupancy ratio of 0.684 (8):0.316 (8).

## Related literature

For details of extraction and synthesis of gossypol and its derivatives, see: Kenar (2006 ▶). For synthesis and biological activities, see: Polsky *et al.* (1989 ▶); Radloff *et al.* (1985 ▶). For formation of crystalline forms of inclusion compounds, see: Ibragimov & Talipov (1999 ▶, 2004 ▶).
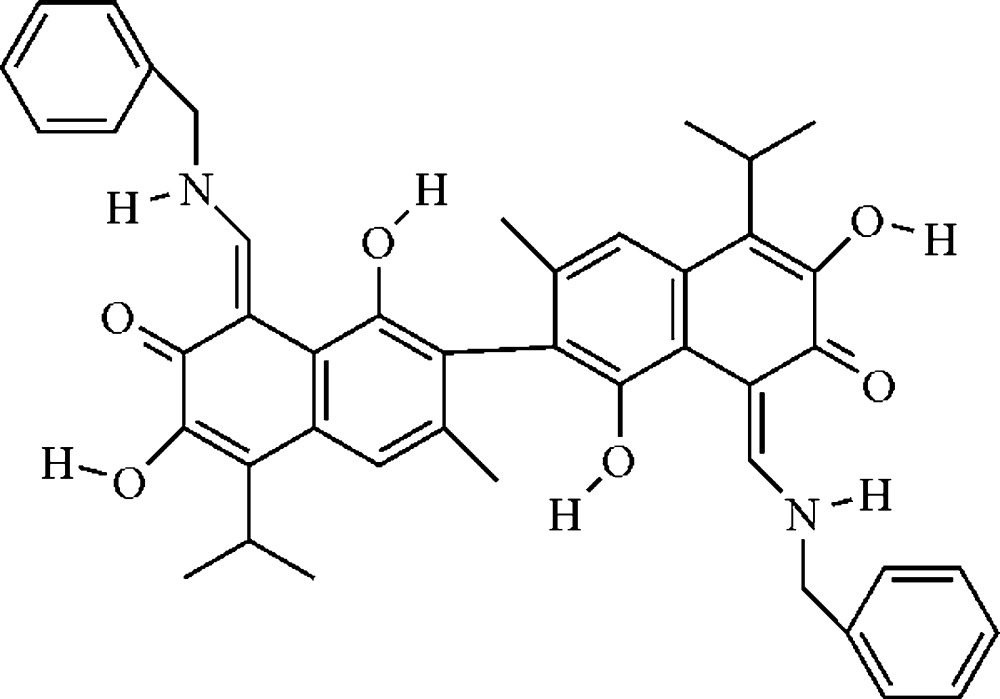


## Experimental

### 

#### Crystal data

C_44_H_44_N_2_O_6_
*M* *_r_* = 696.81
Monoclinic, 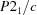

*a* = 9.8118 (8) Å
*b* = 29.014 (2) Å
*c* = 25.5854 (17) Å
β = 90.196 (6)°
*V* = 7283.6 (9) Å^3^
*Z* = 8
Cu *K*α radiation
μ = 0.68 mm^−1^
*T* = 293 K
0.6 × 0.3 × 0.2 mm

#### Data collection

Oxford Diffraction Xcalibur Ruby diffractometer
Absorption correction: multi-scan (*CrysAlis PRO*; Oxford Diffraction, 2009 ▶) *T*_min_ = 0.66, *T*_max_ = 0.88
54670 measured reflections
15043 independent reflections
7763 reflections with *I* > 2σ(*I*)
*R*_int_ = 0.049

#### Refinement

*R*[*F* ^2^ > 2σ(*F* ^2^)] = 0.062
*wR*(*F* ^2^) = 0.191
*S* = 0.94
15043 reflections
1001 parameters
3 restraints
H atoms treated by a mixture of independent and constrained refinement
Δρ_max_ = 0.22 e Å^−3^
Δρ_min_ = −0.26 e Å^−3^

### 

Data collection: *CrysAlis PRO* (Oxford Diffraction, 2009 ▶); cell refinement: *CrysAlis PRO*; data reduction: *CrysAlis PRO*; program(s) used to solve structure: *SHELXS97* (Sheldrick, 2008 ▶); program(s) used to refine structure: *SHELXL97* (Sheldrick, 2008 ▶); molecular graphics: *XP* (Bruker, 1998 ▶); software used to prepare material for publication: *SHELXL97*.

## Supplementary Material

Crystal structure: contains datablock(s) I, New_Global_Publ_Block. DOI: 10.1107/S1600536813027281/xu5738sup1.cif

Structure factors: contains datablock(s) I. DOI: 10.1107/S1600536813027281/xu5738Isup2.hkl

Additional supplementary materials:  crystallographic information; 3D view; checkCIF report

## Figures and Tables

**Table 1:** Hydrogen-bond geometry (Å, °)

*D*—H⋯*A*	*D*—H	H⋯*A*	*D*⋯*A*	*D*—H⋯*A*
N1*A*—H1*AN*⋯O3*A*	0.86	1.79	2.494 (3)	138
N1*B*—H1*BN*⋯O3*B*	0.86	1.85	2.531 (3)	135
N2*A*—H2*AN*⋯O7*A*	0.86	1.93	2.584 (3)	132
N2*B*—H2*BN*⋯O7*B*	0.86	1.89	2.561 (3)	134
O1*A*—H1*A*⋯O7*B* ^i^	0.65 (2)	2.24 (3)	2.778 (3)	141 (3)
O1*B*—H1*B*⋯O7*A* ^ii^	0.77 (3)	2.33 (3)	2.846 (2)	125 (3)
O4*A*—H4*AB*⋯O3*A*	0.74 (3)	2.05 (3)	2.588 (3)	130 (3)
O4*B*—H4*BC*⋯O3*B*	0.83 (4)	1.98 (4)	2.568 (4)	126 (4)
O5*A*—H5*A*⋯O3*B*	0.76 (3)	2.10 (3)	2.714 (3)	138 (3)
O5*B*—H5*B*⋯O3*A*	0.81 (3)	1.98 (3)	2.645 (3)	139 (3)
O8*A*—H8*A*⋯O1*B* ^iii^	0.92 (5)	2.45 (5)	3.290 (3)	151 (4)
O8*A*—H8*A*⋯O7*A*	0.92 (5)	1.95 (5)	2.592 (3)	126 (4)
O8*B*—H8*B*⋯O1*A* ^iv^	0.86 (5)	2.40 (5)	3.183 (3)	151 (4)
O8*B*—H8*B*⋯O7*B*	0.86 (5)	1.93 (5)	2.577 (3)	130 (4)

Symmetry codes: (i) 
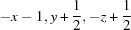
; (ii) 
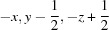
; (iii) 
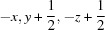
; (iv) 
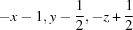
.

**Fig. 1.**
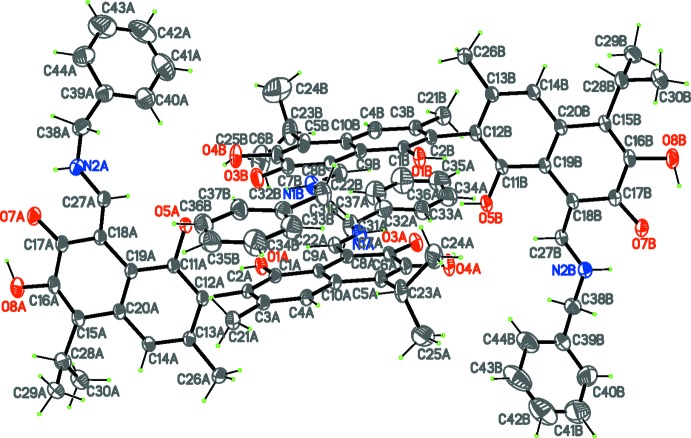
The molecular structure of title compound, with the atom-numbering scheme. Displacement ellipsoids are drawn at the 50% probability level.

**Fig. 2.**
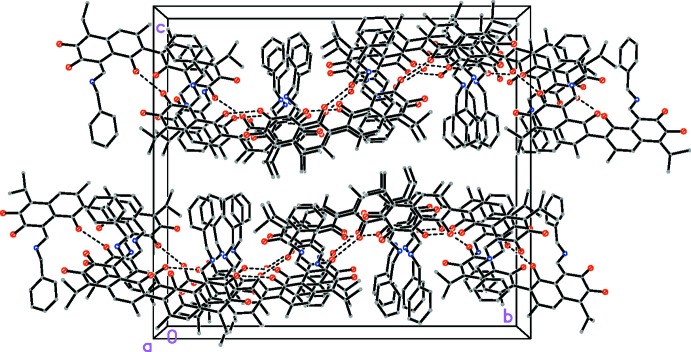
A packing diagram for title compound.

## supplementary crystallographic information

### 1. Comment

Bis-benzylaminogossypol (2,2'-bis(1,6-dihydroxy,-5-isopropyl 8-methine-aminobenzyl-3-methylnaphthalene-7-one), C_44_H_44_O_6_N_2_) is Shiff base derivative of gossypol, a phenolic pigment extracted from cotton seeds (Kenar, 2006). Gossypol demonstrates wide range of biological activities (Polsky *et al.*, 1989: Radloff *et al.*, 1985) and versatile host properties (Ibragimov & Talipov, 1999; 2004). Herein, we describe the crystal structure of bis-benzylaminogossypol. The asymmetric unit of the crystal structure consists of two independent molecules A and B which are linked by a pair of strong O5A—H···O3B and O5B—H···O3A hydrogen bonds and the molecules form a dimer. Molecules of the title compound is racemic and in the enamine tautomer form. The naphthyl moieties of both molecules are planar and nearly perpendicular, the dihedral angles between their least-squares planes are 83.36 (5)° and 89.04 (5)° in the molecules A and B (Fig.1), respectively. The tilt angle between the benzyl rings and corresponding naphtyl moiety are different: (C1A—C10A)/(C31A—C37A) - 9.14 (16)°; (C11A—C20A)/(C38A—C44A) -52.66 (11)°; (C1B—C10B)/(C31B—C37B) -4.41 (14)°; (C11B—C20B)/(C38B—C44B) -65.51 (11)°. Isopropyl group (to C15A) are disordered. In the crystal structure (Fig.2), the dimers of title compound are linked through O1A—H···O7B, O1B—H···O7A, O8A—H···O1B and O8B—H···O1A hydrogen bonds into layers parallel to (0 0 1) (Table 1).

### 2. Experimental

Gossypol was obtained from the Experimental Plant of the Institute of Bioorganic Chemistry, Academy of Sciences of Uzbekistan where it was produced from by-products of the cottonseed oil industry. To prepare the Schiff base compound, gossypol was mixed with benzylamine in a 1:3 molar ratio in the acetone. This reaction solution was allowed to stand in the dark for 3 days, during which crystalline precipitate formed within solution. The precipitate was recovered by filtration and air-dried. Suitable crystals were selected from the precipitate and were used for diffraction without additional recrystallization.

### 3. Refinement

The H atoms attached to oxygen atoms were found from the difference Fourier maps and were refined isotropically, other H atoms were placed in geometrically idealized positions (N—H = 0.86, C—H = 0.93–0.96 Å, and treated as riding on their parent atoms with *U*_iso_(H) = 1.2*U*_eq_(C,*N*) and 1.5*U*_eq_(C). One of isopropyl group is disordered over two positions, occupancies were refined to 0.684 (8):0.316 (8).

### Crystal data

**Table:**

C_44_H_44_N_2_O_6_	*F*(000) = 2960
*M**_r_* = 696.81	*D*_x_ = 1.271 Mg m^−^^3^*D*_m_ = 1.271 Mg m^−^^3^*D*_m_ measured by not measured
Monoclinic, *P*2_1_/*c*	Cu *K*α radiation, λ = 1.54184 Å
*a* = 9.8118 (8) Å	Cell parameters from 3347 reflections
*b* = 29.014 (2) Å	θ = 3.5–36.0°
*c* = 25.5854 (17) Å	µ = 0.68 mm^−^^1^
β = 90.196 (6)°	*T* = 293 K
*V* = 7283.6 (9) Å^3^	Prism, brown
*Z* = 8	0.6 × 0.3 × 0.2 mm

### Data collection

**Table:**

Oxford Diffraction Xcalibur Ruby diffractometer	15043 independent reflections
Radiation source: fine-focus sealed tube	7763 reflections with *I* > 2σ(*I*)
Graphite monochromator	*R*_int_ = 0.049
Detector resolution: 10.2576 pixels mm^-1^	θ_max_ = 76.8°, θ_min_ = 3.5°
ω scans	*h* = −11→12
Absorption correction: multi-scan (*CrysAlis PRO*; Oxford Diffraction, 2009)	*k* = −33→36
*T*_min_ = 0.66, *T*_max_ = 0.88	*l* = −31→31
54670 measured reflections	

### Refinement

**Table:**

Refinement on *F*^2^	Secondary atom site location: difference Fourier map
Least-squares matrix: full	Hydrogen site location: inferred from neighbouring sites
*R*[*F*^2^ > 2σ(*F*^2^)] = 0.062	H atoms treated by a mixture of independent and constrained refinement
*wR*(*F*^2^) = 0.191	*w* = 1/[σ^2^(*F*_o_^2^) + (0.106*P*)^2^] where *P* = (*F*_o_^2^ + 2*F*_c_^2^)/3
*S* = 0.94	(Δ/σ)_max_ = 0.002
15043 reflections	Δρ_max_ = 0.22 e Å^−^^3^
1001 parameters	Δρ_min_ = −0.26 e Å^−^^3^
3 restraints	Extinction correction: *SHELXL97* (Sheldrick, 2008), Fc^*^=kFc[1+0.001xFc^2^λ^3^/sin(2θ)]^-1/4^
Primary atom site location: structure-invariant direct methods	Extinction coefficient: 0.00014 (3)

### Special details

**Table:**

Geometry. All e.s.d.'s (except the e.s.d. in the dihedral angle between two l.s. planes) are estimated using the full covariance matrix. The cell e.s.d.'s are taken into account individually in the estimation of e.s.d.'s in distances, angles and torsion angles; correlations between e.s.d.'s in cell parameters are only used when they are defined by crystal symmetry. An approximate (isotropic) treatment of cell e.s.d.'s is used for estimating e.s.d.'s involving l.s. planes.
Refinement. Refinement of *F*^2^ against ALL reflections. The weighted *R*-factor *wR* and goodness of fit *S* are based on *F*^2^, conventional *R*-factors *R* are based on *F*, with *F* set to zero for negative *F*^2^. The threshold expression of *F*^2^ > σ(*F*^2^) is used only for calculating *R*-factors(gt) *etc*. and is not relevant to the choice of reflections for refinement. *R*-factors based on *F*^2^ are statistically about twice as large as those based on *F*, and *R*- factors based on ALL data will be even larger.

### Fractional atomic coordinates and isotropic or equivalent isotropic displacement parameters (Å^2^)

**Table:**

	*x*	*y*	*z*	*U*_iso_*/*U*_eq_	Occ. (<1)
O1A	−0.3398 (2)	0.50674 (7)	0.30051 (8)	0.0599 (5)	
O3A	−0.51864 (19)	0.35070 (6)	0.23365 (8)	0.0662 (5)	
O4A	−0.3897 (3)	0.28774 (7)	0.28570 (10)	0.0758 (7)	
O5A	−0.0079 (2)	0.54677 (7)	0.30415 (8)	0.0600 (5)	
O7A	0.1016 (2)	0.71837 (6)	0.31337 (8)	0.0670 (5)	
O8A	−0.0477 (2)	0.75200 (6)	0.38755 (10)	0.0760 (6)	
N1A	−0.5755 (2)	0.43231 (8)	0.21212 (9)	0.0612 (6)	
H1AN	−0.5811	0.4032	0.2060	0.073*	
N2A	0.2222 (2)	0.65010 (8)	0.26806 (9)	0.0620 (6)	
H2AN	0.2113	0.6795	0.2693	0.074*	
C1A	−0.2683 (2)	0.47309 (8)	0.32578 (9)	0.0449 (5)	
C2A	−0.1730 (2)	0.48497 (8)	0.36297 (9)	0.0442 (5)	
C3A	−0.1000 (2)	0.44951 (8)	0.38860 (9)	0.0463 (6)	
C4A	−0.1287 (2)	0.40481 (8)	0.37605 (10)	0.0484 (6)	
H4	−0.0801	0.3817	0.3930	0.058*	
C5A	−0.2540 (3)	0.34321 (8)	0.32885 (10)	0.0538 (6)	
C6A	−0.3536 (3)	0.33248 (8)	0.29462 (11)	0.0549 (7)	
C7A	−0.4285 (3)	0.36623 (9)	0.26604 (10)	0.0522 (6)	
C8A	−0.4036 (2)	0.41362 (8)	0.27448 (9)	0.0469 (6)	
C9A	−0.3011 (2)	0.42680 (8)	0.31242 (9)	0.0422 (5)	
C10A	−0.2273 (2)	0.39184 (8)	0.33906 (9)	0.0452 (6)	
C11A	−0.0709 (2)	0.56317 (8)	0.34810 (9)	0.0449 (5)	
C12A	−0.1535 (2)	0.53450 (8)	0.37748 (9)	0.0456 (6)	
C13A	−0.2189 (3)	0.55251 (8)	0.42227 (10)	0.0507 (6)	
C14A	−0.2018 (3)	0.59822 (8)	0.43407 (10)	0.0527 (6)	
H14A	−0.2456	0.6100	0.4634	0.063*	
C15A	−0.1140 (3)	0.67728 (8)	0.41650 (11)	0.0537 (6)	
C16A	−0.0481 (3)	0.70493 (8)	0.38213 (11)	0.0550 (7)	
C17A	0.0340 (3)	0.68878 (8)	0.33948 (10)	0.0524 (6)	
C18A	0.0404 (3)	0.64017 (8)	0.33049 (9)	0.0472 (6)	
C19A	−0.0487 (2)	0.61000 (8)	0.36051 (9)	0.0452 (5)	
C20A	−0.1213 (3)	0.62822 (8)	0.40407 (10)	0.0478 (6)	
C21A	0.0057 (3)	0.46067 (9)	0.42926 (11)	0.0617 (7)	
H21A	0.0788	0.4776	0.4134	0.093*	
H21B	0.0407	0.4326	0.4439	0.093*	
H21C	−0.0347	0.4789	0.4564	0.093*	
C22A	−0.4844 (3)	0.44502 (9)	0.24534 (10)	0.0574 (7)	
H22A	−0.4708	0.4764	0.2505	0.069*	
C23A	−0.1769 (4)	0.30519 (9)	0.35660 (13)	0.0762 (9)	
H23A	−0.0982	0.3197	0.3736	0.091*	
C24A	−0.1200 (4)	0.26728 (11)	0.32034 (16)	0.0973 (12)	
H24A	−0.1934	0.2482	0.3081	0.146*	
H24B	−0.0557	0.2487	0.3393	0.146*	
H24C	−0.0755	0.2813	0.2910	0.146*	
C25A	−0.2630 (6)	0.28451 (14)	0.39965 (15)	0.1304 (18)	
H25A	−0.2812	0.3075	0.4256	0.196*	
H25B	−0.2150	0.2592	0.4154	0.196*	
H25C	−0.3476	0.2737	0.3852	0.196*	
C26A	−0.3089 (3)	0.52265 (9)	0.45625 (11)	0.0675 (8)	
H26A	−0.3893	0.5142	0.4371	0.101*	
H26B	−0.3343	0.5395	0.4870	0.101*	
H26C	−0.2600	0.4953	0.4662	0.101*	
C27A	0.1424 (3)	0.62403 (9)	0.29687 (10)	0.0519 (6)	
H27A	0.1546	0.5923	0.2947	0.062*	
C28A	−0.1746 (4)	0.69730 (10)	0.46614 (13)	0.0785 (9)	
H28A	−0.1450	0.7295	0.4647	0.094*	0.684 (8)
H28C	−0.2481	0.6747	0.4701	0.094*	0.316 (8)
C29A	−0.1023 (9)	0.6797 (3)	0.5133 (3)	0.076 (2)	0.684 (8)
H29A	−0.1295	0.6484	0.5198	0.114*	0.684 (8)
H29B	−0.1252	0.6985	0.5429	0.114*	0.684 (8)
H29C	−0.0057	0.6808	0.5076	0.114*	0.684 (8)
C30A	−0.3198 (5)	0.7014 (2)	0.4704 (2)	0.0801 (19)	0.684 (8)
H30A	−0.3613	0.6721	0.4635	0.120*	0.684 (8)
H30B	−0.3523	0.7236	0.4455	0.120*	0.684 (8)
H30C	−0.3429	0.7113	0.5051	0.120*	0.684 (8)
C29C	−0.2682 (13)	0.7406 (4)	0.4553 (5)	0.096 (5)	0.316 (8)
H29G	−0.3045	0.7388	0.4204	0.143*	0.316 (8)
H29H	−0.2152	0.7683	0.4588	0.143*	0.316 (8)
H29I	−0.3417	0.7411	0.4799	0.143*	0.316 (8)
C30C	−0.114 (3)	0.6915 (10)	0.5192 (6)	0.181 (15)	0.316 (8)
H30G	−0.0640	0.6632	0.5206	0.271*	0.316 (8)
H30H	−0.1859	0.6908	0.5447	0.271*	0.316 (8)
H30I	−0.0544	0.7169	0.5265	0.271*	0.316 (8)
C31A	−0.6679 (4)	0.46109 (12)	0.18448 (16)	0.0955 (12)	
H31A	−0.6165	0.4855	0.1677	0.115*	
H31B	−0.7287	0.4755	0.2095	0.115*	
C32A	−0.7541 (3)	0.43661 (13)	0.14295 (14)	0.0817 (9)	
C33A	−0.7725 (4)	0.38898 (14)	0.14119 (16)	0.0953 (12)	
H33A	−0.7268	0.3698	0.1645	0.114*	
C34A	−0.8578 (5)	0.37130 (18)	0.10505 (19)	0.1115 (14)	
H34A	−0.8719	0.3396	0.1054	0.134*	
C35A	−0.9227 (5)	0.3955 (2)	0.0693 (2)	0.1365 (19)	
H35A	−0.9767	0.3814	0.0440	0.164*	
C36A	−0.9066 (7)	0.4430 (2)	0.0712 (2)	0.163 (2)	
H36A	−0.9541	0.4620	0.0482	0.196*	
C37A	−0.8190 (5)	0.46168 (19)	0.10773 (19)	0.1387 (19)	
H37A	−0.8051	0.4934	0.1076	0.166*	
C38A	0.3275 (3)	0.63183 (12)	0.23430 (12)	0.0745 (9)	
H38A	0.3579	0.6024	0.2481	0.089*	
H38B	0.4048	0.6527	0.2348	0.089*	
C39A	0.2821 (3)	0.62517 (11)	0.17855 (13)	0.0731 (8)	
C40A	0.1828 (5)	0.59550 (17)	0.16518 (17)	0.1199 (16)	
H40A	0.1386	0.5784	0.1908	0.144*	
C41A	0.1461 (6)	0.5904 (2)	0.1129 (2)	0.149 (2)	
H41A	0.0764	0.5702	0.1037	0.179*	
C42A	0.2109 (8)	0.6145 (2)	0.0753 (2)	0.150 (3)	
H42A	0.1872	0.6108	0.0404	0.180*	
C43A	0.3105 (7)	0.6441 (2)	0.0893 (2)	0.156 (2)	
H43A	0.3554	0.6612	0.0639	0.187*	
C44A	0.3451 (5)	0.64900 (15)	0.13937 (16)	0.1105 (14)	
H44A	0.4147	0.6694	0.1480	0.133*	
H1A	−0.319 (3)	0.5272 (9)	0.3076 (10)	0.043 (9)*	
H4AB	−0.437 (3)	0.2912 (11)	0.2635 (13)	0.075 (13)*	
H5A	−0.019 (3)	0.5211 (10)	0.2998 (11)	0.070 (10)*	
H8A	0.009 (6)	0.7604 (18)	0.361 (2)	0.19 (2)*	
O1B	−0.1752 (2)	0.31302 (6)	0.17875 (8)	0.0614 (5)	
O3B	−0.0174 (2)	0.47414 (7)	0.23736 (8)	0.0749 (6)	
O4B	−0.1476 (3)	0.53280 (7)	0.18104 (13)	0.0950 (8)	
O5B	−0.5256 (2)	0.27491 (6)	0.17625 (8)	0.0617 (5)	
O7B	−0.6126 (2)	0.10094 (6)	0.19258 (8)	0.0639 (5)	
O8B	−0.4496 (2)	0.06113 (6)	0.12801 (10)	0.0781 (7)	
N1B	0.0548 (2)	0.39295 (8)	0.26071 (9)	0.0612 (6)	
H1BN	0.0598	0.4220	0.2669	0.073*	
N2B	−0.7309 (2)	0.17165 (8)	0.23157 (9)	0.0591 (6)	
H2BN	−0.7222	0.1422	0.2313	0.071*	
C1B	−0.2491 (3)	0.34500 (8)	0.15120 (10)	0.0482 (6)	
C2B	−0.3468 (3)	0.33002 (8)	0.11598 (9)	0.0476 (6)	
C3B	−0.4201 (3)	0.36252 (9)	0.08731 (10)	0.0530 (6)	
C4B	−0.3928 (3)	0.40850 (9)	0.09530 (11)	0.0610 (7)	
H4B	−0.4417	0.4300	0.0759	0.073*	
C5B	−0.2728 (3)	0.47405 (9)	0.13804 (13)	0.0672 (8)	
C6B	−0.1792 (3)	0.48738 (9)	0.17298 (13)	0.0680 (8)	
C7B	−0.1010 (3)	0.45603 (9)	0.20466 (11)	0.0582 (7)	
C8B	−0.1189 (2)	0.40818 (8)	0.19770 (10)	0.0483 (6)	
C9B	−0.2192 (2)	0.39209 (8)	0.16036 (10)	0.0468 (6)	
C10B	−0.2958 (3)	0.42474 (8)	0.13102 (11)	0.0540 (6)	
C11B	−0.4478 (2)	0.25358 (8)	0.13916 (9)	0.0464 (6)	
C12B	−0.3616 (3)	0.27949 (8)	0.10783 (10)	0.0479 (6)	
C13B	−0.2832 (3)	0.25708 (9)	0.06921 (10)	0.0516 (6)	
C14B	−0.2905 (3)	0.20996 (8)	0.06589 (10)	0.0531 (6)	
H14B	−0.2385	0.1952	0.0406	0.064*	
C15B	−0.3681 (3)	0.13275 (8)	0.09532 (10)	0.0499 (6)	
C16B	−0.4483 (3)	0.10820 (8)	0.12741 (11)	0.0540 (6)	
C17B	−0.5413 (3)	0.12858 (8)	0.16441 (10)	0.0496 (6)	
C18B	−0.5507 (2)	0.17727 (8)	0.16775 (9)	0.0461 (6)	
C19B	−0.4589 (2)	0.20484 (8)	0.13539 (9)	0.0452 (5)	
C20B	−0.3724 (2)	0.18272 (8)	0.09873 (9)	0.0463 (6)	
C21B	−0.5251 (3)	0.34706 (11)	0.04815 (12)	0.0731 (9)	
H21D	−0.4824	0.3283	0.0220	0.110*	
H21F	−0.5656	0.3735	0.0318	0.110*	
H21E	−0.5943	0.3295	0.0656	0.110*	
C22B	−0.0336 (3)	0.37892 (9)	0.22677 (11)	0.0568 (7)	
H22B	−0.0413	0.3473	0.2212	0.068*	
C23B	−0.3500 (4)	0.50935 (11)	0.10536 (19)	0.1092 (14)	
H23B	−0.4302	0.4931	0.0918	0.131*	
C24B	−0.2709 (7)	0.52298 (19)	0.0589 (2)	0.181 (3)	
H24D	−0.1836	0.5346	0.0697	0.272*	
H24E	−0.3192	0.5466	0.0401	0.272*	
H24F	−0.2584	0.4967	0.0366	0.272*	
C25B	−0.4062 (5)	0.55037 (14)	0.1370 (3)	0.165 (2)	
H25D	−0.4431	0.5394	0.1694	0.247*	
H25E	−0.4766	0.5654	0.1172	0.247*	
H25F	−0.3339	0.5718	0.1440	0.247*	
C26B	−0.1927 (3)	0.28431 (10)	0.03317 (11)	0.0665 (8)	
H26F	−0.2479	0.3035	0.0110	0.100*	
H26D	−0.1402	0.2635	0.0121	0.100*	
H26E	−0.1324	0.3033	0.0535	0.100*	
C27B	−0.6516 (3)	0.19577 (9)	0.20076 (10)	0.0515 (6)	
H27B	−0.6628	0.2276	0.2007	0.062*	
C28B	−0.2757 (3)	0.11051 (9)	0.05479 (11)	0.0603 (7)	
H28B	−0.1975	0.1312	0.0508	0.072*	
C29B	−0.3490 (4)	0.11025 (11)	0.00226 (12)	0.0783 (9)	
H29E	−0.4227	0.0885	0.0033	0.117*	
H29D	−0.2861	0.1016	−0.0247	0.117*	
H29F	−0.3842	0.1405	−0.0049	0.117*	
C30B	−0.2174 (3)	0.06336 (10)	0.06733 (13)	0.0739 (9)	
H30D	−0.1935	0.0620	0.1037	0.111*	
H30E	−0.1377	0.0581	0.0466	0.111*	
H30F	−0.2843	0.0401	0.0597	0.111*	
C31B	0.1449 (3)	0.36288 (11)	0.28869 (14)	0.0822 (10)	
H31C	0.2016	0.3467	0.2637	0.099*	
H31D	0.0910	0.3401	0.3070	0.099*	
C32B	0.2354 (3)	0.38680 (11)	0.32731 (12)	0.0679 (8)	
C33B	0.3082 (4)	0.36054 (14)	0.36087 (14)	0.0946 (11)	
H33B	0.2974	0.3287	0.3601	0.114*	
C34B	0.3972 (5)	0.37956 (18)	0.39591 (17)	0.1196 (15)	
H34B	0.4470	0.3608	0.4184	0.144*	
C35B	0.4129 (4)	0.42655 (17)	0.39777 (15)	0.0977 (13)	
H35B	0.4726	0.4399	0.4217	0.117*	
C36B	0.3420 (4)	0.45267 (14)	0.36510 (16)	0.0928 (11)	
H36B	0.3528	0.4845	0.3663	0.111*	
C37B	0.2527 (3)	0.43390 (12)	0.32942 (13)	0.0789 (9)	
H37B	0.2042	0.4529	0.3068	0.095*	
C38B	−0.8325 (3)	0.19135 (11)	0.26616 (11)	0.0678 (8)	
H38C	−0.9160	0.1736	0.2631	0.081*	
H38D	−0.8522	0.2226	0.2549	0.081*	
C39B	−0.7893 (3)	0.19230 (11)	0.32213 (13)	0.0679 (8)	
C40B	−0.8537 (6)	0.16527 (16)	0.35764 (17)	0.1373 (19)	
H40B	−0.9230	0.1456	0.3467	0.165*	
C41B	−0.8171 (10)	0.1668 (2)	0.4103 (2)	0.195 (3)	
H41B	−0.8623	0.1484	0.4344	0.234*	
C42B	−0.7170 (8)	0.1947 (3)	0.4261 (3)	0.167 (3)	
H42B	−0.6909	0.1951	0.4610	0.200*	
C43B	−0.6554 (6)	0.2217 (3)	0.3926 (3)	0.160 (3)	
H43B	−0.5874	0.2416	0.4042	0.192*	
C44B	−0.6911 (4)	0.22078 (17)	0.33914 (18)	0.1212 (17)	
H44B	−0.6465	0.2400	0.3157	0.145*	
H1B	−0.209 (3)	0.2892 (11)	0.1740 (12)	0.081 (11)*	
H4BC	−0.090 (5)	0.5312 (16)	0.2050 (18)	0.14 (2)*	
H5B	−0.509 (3)	0.3021 (10)	0.1796 (11)	0.063 (9)*	
H8B	−0.512 (5)	0.0569 (16)	0.1514 (19)	0.17 (2)*	

### Atomic displacement parameters (Å^2^)

**Table:**

	*U*^11^	*U*^22^	*U*^33^	*U*^12^	*U*^13^	*U*^23^
O1A	0.0747 (13)	0.0302 (11)	0.0748 (13)	0.0014 (9)	−0.0184 (10)	−0.0040 (9)
O3A	0.0655 (12)	0.0554 (12)	0.0777 (13)	−0.0064 (9)	−0.0126 (10)	−0.0225 (10)
O4A	0.0997 (18)	0.0342 (11)	0.0932 (17)	−0.0114 (10)	−0.0250 (15)	−0.0085 (10)
O5A	0.0788 (13)	0.0384 (11)	0.0629 (12)	−0.0152 (9)	0.0150 (10)	−0.0141 (9)
O7A	0.0913 (14)	0.0373 (10)	0.0722 (12)	−0.0152 (9)	−0.0054 (11)	0.0070 (9)
O8A	0.0984 (17)	0.0290 (10)	0.1005 (17)	−0.0028 (10)	0.0002 (14)	−0.0069 (10)
N1A	0.0600 (14)	0.0557 (14)	0.0679 (15)	0.0071 (11)	−0.0175 (12)	−0.0110 (11)
N2A	0.0710 (15)	0.0531 (14)	0.0619 (14)	−0.0147 (11)	0.0034 (12)	0.0032 (11)
C1A	0.0496 (14)	0.0322 (12)	0.0529 (14)	0.0008 (10)	0.0018 (12)	−0.0004 (10)
C2A	0.0508 (14)	0.0316 (12)	0.0502 (13)	−0.0063 (10)	0.0029 (11)	−0.0014 (10)
C3A	0.0511 (14)	0.0375 (13)	0.0504 (14)	−0.0025 (10)	−0.0010 (11)	−0.0007 (10)
C4A	0.0534 (15)	0.0339 (13)	0.0577 (15)	0.0007 (10)	−0.0036 (12)	0.0030 (10)
C5A	0.0647 (16)	0.0337 (13)	0.0630 (16)	−0.0017 (11)	−0.0029 (14)	−0.0017 (11)
C6A	0.0656 (17)	0.0340 (13)	0.0650 (17)	−0.0052 (11)	0.0005 (14)	−0.0085 (11)
C7A	0.0553 (15)	0.0452 (15)	0.0559 (15)	−0.0039 (11)	0.0005 (13)	−0.0116 (12)
C8A	0.0490 (14)	0.0406 (13)	0.0510 (14)	0.0014 (10)	0.0031 (11)	−0.0057 (10)
C9A	0.0436 (13)	0.0348 (12)	0.0483 (13)	−0.0016 (9)	0.0025 (11)	−0.0056 (10)
C10A	0.0498 (14)	0.0343 (12)	0.0516 (14)	−0.0036 (10)	−0.0013 (11)	−0.0016 (10)
C11A	0.0512 (14)	0.0355 (13)	0.0480 (13)	−0.0034 (10)	−0.0029 (11)	−0.0025 (10)
C12A	0.0526 (14)	0.0328 (12)	0.0513 (14)	−0.0051 (10)	−0.0054 (12)	−0.0021 (10)
C13A	0.0610 (16)	0.0375 (14)	0.0536 (14)	−0.0044 (11)	0.0007 (12)	−0.0008 (11)
C14A	0.0672 (17)	0.0371 (13)	0.0540 (15)	−0.0012 (11)	0.0036 (13)	−0.0052 (11)
C15A	0.0597 (16)	0.0374 (14)	0.0641 (16)	0.0016 (11)	−0.0076 (13)	−0.0078 (11)
C16A	0.0663 (17)	0.0295 (13)	0.0690 (17)	−0.0013 (11)	−0.0144 (14)	−0.0037 (11)
C17A	0.0663 (17)	0.0336 (13)	0.0573 (15)	−0.0062 (11)	−0.0098 (13)	0.0025 (11)
C18A	0.0565 (15)	0.0374 (13)	0.0476 (13)	−0.0053 (11)	−0.0088 (12)	0.0005 (10)
C19A	0.0514 (14)	0.0349 (13)	0.0493 (14)	−0.0026 (10)	−0.0093 (11)	0.0014 (10)
C20A	0.0551 (15)	0.0337 (13)	0.0545 (14)	−0.0004 (10)	−0.0063 (12)	−0.0023 (10)
C21A	0.0690 (18)	0.0488 (16)	0.0674 (18)	−0.0068 (13)	−0.0127 (15)	−0.0020 (13)
C22A	0.0607 (17)	0.0467 (16)	0.0649 (17)	0.0000 (12)	−0.0036 (14)	−0.0068 (13)
C23A	0.099 (2)	0.0349 (15)	0.094 (2)	0.0027 (14)	−0.0294 (19)	−0.0025 (14)
C24A	0.106 (3)	0.048 (2)	0.138 (3)	0.0174 (18)	0.002 (2)	−0.004 (2)
C25A	0.234 (6)	0.078 (3)	0.079 (3)	0.025 (3)	0.007 (3)	0.019 (2)
C26A	0.087 (2)	0.0476 (16)	0.0678 (18)	−0.0081 (14)	0.0175 (16)	−0.0005 (13)
C27A	0.0596 (16)	0.0432 (14)	0.0528 (15)	−0.0100 (12)	−0.0082 (13)	0.0021 (11)
C28A	0.103 (3)	0.0453 (17)	0.088 (2)	0.0086 (16)	0.018 (2)	−0.0123 (15)
C29A	0.090 (5)	0.080 (4)	0.059 (4)	−0.011 (3)	0.009 (3)	−0.024 (3)
C30A	0.069 (3)	0.077 (4)	0.094 (4)	0.009 (3)	−0.011 (3)	−0.019 (3)
C29C	0.088 (9)	0.083 (10)	0.116 (10)	0.035 (7)	−0.001 (7)	−0.040 (8)
C30C	0.16 (2)	0.28 (4)	0.103 (15)	0.10 (2)	−0.037 (13)	−0.103 (17)
C31A	0.096 (3)	0.073 (2)	0.118 (3)	−0.0041 (19)	−0.034 (2)	−0.001 (2)
C32A	0.072 (2)	0.093 (3)	0.081 (2)	−0.0022 (19)	−0.0079 (18)	0.002 (2)
C33A	0.087 (3)	0.099 (3)	0.101 (3)	−0.024 (2)	−0.012 (2)	−0.017 (2)
C34A	0.103 (3)	0.122 (4)	0.109 (3)	−0.021 (3)	0.002 (3)	−0.021 (3)
C35A	0.122 (4)	0.180 (6)	0.107 (4)	−0.017 (4)	−0.028 (3)	−0.019 (4)
C36A	0.183 (6)	0.161 (6)	0.145 (5)	0.000 (5)	−0.079 (4)	0.014 (4)
C37A	0.152 (4)	0.135 (4)	0.129 (4)	−0.005 (3)	−0.070 (4)	0.012 (3)
C38A	0.0644 (19)	0.089 (2)	0.070 (2)	−0.0120 (16)	0.0059 (16)	0.0070 (17)
C39A	0.075 (2)	0.074 (2)	0.071 (2)	0.0008 (16)	0.0025 (17)	0.0031 (16)
C40A	0.122 (4)	0.147 (4)	0.090 (3)	−0.043 (3)	−0.009 (3)	−0.016 (3)
C41A	0.148 (5)	0.171 (6)	0.128 (4)	−0.005 (4)	−0.054 (4)	−0.041 (4)
C42A	0.211 (7)	0.162 (6)	0.075 (3)	0.048 (5)	−0.025 (4)	−0.011 (4)
C43A	0.212 (7)	0.171 (6)	0.086 (4)	0.019 (5)	−0.009 (4)	0.030 (4)
C44A	0.141 (4)	0.110 (3)	0.081 (3)	−0.015 (3)	−0.001 (3)	0.026 (2)
O1B	0.0779 (13)	0.0323 (10)	0.0738 (13)	−0.0018 (9)	−0.0263 (10)	−0.0036 (9)
O3B	0.0786 (14)	0.0537 (12)	0.0922 (15)	−0.0171 (10)	−0.0036 (12)	−0.0267 (11)
O4B	0.112 (2)	0.0351 (12)	0.137 (2)	−0.0174 (12)	−0.0101 (19)	−0.0076 (13)
O5B	0.0749 (13)	0.0356 (11)	0.0748 (13)	−0.0057 (9)	0.0098 (10)	−0.0152 (9)
O7B	0.0749 (13)	0.0391 (10)	0.0778 (13)	−0.0067 (9)	0.0053 (11)	−0.0017 (9)
O8B	0.0873 (16)	0.0312 (10)	0.1159 (19)	0.0006 (9)	0.0212 (14)	−0.0076 (10)
N1B	0.0565 (13)	0.0566 (14)	0.0703 (15)	−0.0042 (11)	−0.0132 (12)	−0.0166 (11)
N2B	0.0639 (14)	0.0479 (13)	0.0655 (14)	−0.0018 (10)	0.0047 (12)	−0.0057 (11)
C1B	0.0580 (15)	0.0344 (13)	0.0521 (14)	0.0001 (11)	−0.0017 (12)	−0.0023 (10)
C2B	0.0589 (15)	0.0339 (13)	0.0501 (14)	−0.0030 (10)	−0.0030 (12)	−0.0037 (10)
C3B	0.0570 (15)	0.0451 (15)	0.0570 (15)	−0.0003 (11)	−0.0072 (13)	0.0004 (11)
C4B	0.0673 (18)	0.0450 (16)	0.0706 (18)	0.0029 (13)	−0.0094 (15)	0.0078 (13)
C5B	0.0669 (18)	0.0341 (14)	0.101 (2)	−0.0012 (12)	0.0011 (17)	0.0011 (14)
C6B	0.075 (2)	0.0330 (14)	0.096 (2)	−0.0094 (13)	0.0088 (18)	−0.0084 (14)
C7B	0.0585 (16)	0.0462 (15)	0.0699 (18)	−0.0091 (12)	0.0072 (14)	−0.0109 (13)
C8B	0.0484 (14)	0.0404 (14)	0.0562 (15)	−0.0051 (10)	0.0031 (12)	−0.0104 (11)
C9B	0.0506 (14)	0.0341 (13)	0.0556 (14)	−0.0020 (10)	0.0046 (12)	−0.0048 (10)
C10B	0.0564 (16)	0.0340 (13)	0.0717 (17)	−0.0005 (11)	0.0006 (14)	−0.0019 (12)
C11B	0.0530 (14)	0.0364 (13)	0.0496 (14)	0.0032 (10)	−0.0082 (12)	−0.0097 (10)
C12B	0.0557 (15)	0.0368 (13)	0.0511 (14)	−0.0019 (10)	−0.0127 (12)	−0.0040 (10)
C13B	0.0617 (16)	0.0439 (15)	0.0493 (14)	−0.0033 (11)	−0.0053 (12)	−0.0038 (11)
C14B	0.0624 (16)	0.0440 (15)	0.0528 (15)	−0.0003 (12)	−0.0027 (13)	−0.0100 (11)
C15B	0.0522 (15)	0.0368 (13)	0.0606 (15)	0.0018 (10)	−0.0070 (12)	−0.0111 (11)
C16B	0.0566 (15)	0.0326 (13)	0.0727 (18)	0.0005 (11)	−0.0036 (14)	−0.0083 (12)
C17B	0.0507 (14)	0.0396 (14)	0.0585 (15)	−0.0024 (11)	−0.0072 (12)	−0.0033 (11)
C18B	0.0499 (14)	0.0353 (13)	0.0530 (14)	−0.0010 (10)	−0.0094 (11)	−0.0070 (10)
C19B	0.0510 (14)	0.0365 (13)	0.0480 (13)	0.0009 (10)	−0.0093 (11)	−0.0042 (10)
C20B	0.0494 (14)	0.0403 (13)	0.0492 (13)	−0.0004 (10)	−0.0092 (11)	−0.0082 (10)
C21B	0.079 (2)	0.066 (2)	0.075 (2)	0.0026 (16)	−0.0229 (17)	0.0000 (16)
C22B	0.0565 (16)	0.0476 (16)	0.0664 (17)	−0.0060 (12)	−0.0010 (14)	−0.0139 (13)
C23B	0.130 (3)	0.0405 (19)	0.157 (4)	0.0075 (19)	−0.042 (3)	0.008 (2)
C24B	0.225 (7)	0.156 (5)	0.164 (5)	0.033 (5)	0.007 (5)	0.093 (4)
C25B	0.133 (4)	0.057 (3)	0.304 (8)	0.026 (3)	−0.022 (5)	0.004 (4)
C26B	0.083 (2)	0.0548 (18)	0.0620 (17)	−0.0074 (14)	0.0072 (16)	−0.0031 (13)
C27B	0.0553 (15)	0.0392 (14)	0.0598 (16)	0.0000 (11)	−0.0042 (13)	−0.0066 (11)
C28B	0.0679 (18)	0.0410 (15)	0.0719 (18)	0.0004 (12)	0.0026 (15)	−0.0086 (13)
C29B	0.102 (2)	0.065 (2)	0.068 (2)	0.0100 (17)	0.0082 (18)	−0.0068 (15)
C30B	0.077 (2)	0.0592 (19)	0.086 (2)	0.0146 (15)	−0.0040 (17)	−0.0182 (16)
C31B	0.078 (2)	0.070 (2)	0.098 (3)	−0.0057 (17)	−0.025 (2)	−0.0113 (18)
C32B	0.0563 (17)	0.078 (2)	0.0694 (19)	−0.0081 (15)	−0.0024 (15)	−0.0118 (16)
C33B	0.095 (3)	0.100 (3)	0.089 (3)	−0.010 (2)	−0.030 (2)	0.001 (2)
C34B	0.133 (4)	0.126 (4)	0.099 (3)	−0.017 (3)	−0.044 (3)	0.006 (3)
C35B	0.080 (3)	0.135 (4)	0.078 (2)	−0.024 (2)	−0.015 (2)	−0.022 (2)
C36B	0.083 (2)	0.096 (3)	0.099 (3)	−0.018 (2)	0.003 (2)	−0.026 (2)
C37B	0.066 (2)	0.081 (2)	0.089 (2)	−0.0139 (17)	−0.0082 (18)	−0.0185 (18)
C38B	0.0587 (17)	0.075 (2)	0.0698 (19)	0.0017 (14)	0.0018 (15)	−0.0074 (16)
C39B	0.0688 (19)	0.0627 (19)	0.072 (2)	0.0074 (15)	−0.0046 (16)	−0.0122 (15)
C40B	0.214 (6)	0.112 (4)	0.086 (3)	−0.042 (4)	−0.014 (3)	0.015 (3)
C41B	0.358 (12)	0.143 (6)	0.084 (4)	−0.003 (6)	−0.031 (5)	0.021 (3)
C42B	0.212 (8)	0.175 (7)	0.112 (5)	0.069 (6)	−0.072 (5)	−0.037 (5)
C43B	0.112 (4)	0.237 (9)	0.131 (5)	−0.003 (4)	−0.025 (4)	−0.086 (5)
C44B	0.093 (3)	0.162 (5)	0.109 (3)	−0.031 (3)	−0.001 (3)	−0.055 (3)

### Geometric parameters (Å, º)

**Table:**

O1A—C1A	1.364 (3)	C42A—H42A	0.9300
O1A—H1A	0.65 (2)	C43A—C44A	1.331 (6)
O3A—C7A	1.291 (3)	C43A—H43A	0.9300
O4A—C6A	1.365 (3)	C44A—H44A	0.9300
O4A—H4AB	0.74 (3)	O1B—C1B	1.371 (3)
O5A—C11A	1.370 (3)	O1B—H1B	0.77 (3)
O5A—H5A	0.76 (3)	O3B—C7B	1.282 (3)
O7A—C17A	1.275 (3)	O4B—C6B	1.369 (3)
O8A—C16A	1.373 (3)	O4B—H4BC	0.83 (4)
O8A—H8A	0.92 (5)	O5B—C11B	1.368 (3)
N1A—C22A	1.286 (3)	O5B—H5B	0.81 (3)
N1A—C31A	1.419 (4)	O7B—C17B	1.287 (3)
N1A—H1AN	0.8600	O8B—C16B	1.366 (3)
N2A—C27A	1.316 (3)	O8B—H8B	0.86 (5)
N2A—C38A	1.449 (4)	N1B—C22B	1.291 (3)
N2A—H2AN	0.8600	N1B—C31B	1.432 (4)
C1A—C2A	1.376 (3)	N1B—H1BN	0.8600
C1A—C9A	1.423 (3)	N2B—C27B	1.312 (3)
C2A—C3A	1.413 (3)	N2B—C38B	1.452 (3)
C2A—C12A	1.496 (3)	N2B—H2BN	0.8600
C3A—C4A	1.365 (3)	C1B—C2B	1.383 (3)
C3A—C21A	1.502 (3)	C1B—C9B	1.417 (3)
C4A—C10A	1.403 (3)	C2B—C3B	1.393 (3)
C4A—H4	0.9300	C2B—C12B	1.488 (3)
C5A—C6A	1.347 (4)	C3B—C4B	1.376 (4)
C5A—C10A	1.458 (3)	C3B—C21B	1.503 (4)
C5A—C23A	1.513 (4)	C4B—C10B	1.399 (4)
C6A—C7A	1.425 (4)	C4B—H4B	0.9300
C7A—C8A	1.413 (3)	C5B—C6B	1.336 (4)
C8A—C22A	1.418 (3)	C5B—C10B	1.459 (3)
C8A—C9A	1.447 (3)	C5B—C23B	1.522 (4)
C9A—C10A	1.419 (3)	C6B—C7B	1.438 (4)
C11A—C12A	1.385 (3)	C7B—C8B	1.411 (3)
C11A—C19A	1.412 (3)	C8B—C22B	1.403 (4)
C12A—C13A	1.415 (3)	C8B—C9B	1.447 (3)
C13A—C14A	1.370 (3)	C9B—C10B	1.422 (3)
C13A—C26A	1.514 (3)	C11B—C12B	1.388 (3)
C14A—C20A	1.405 (3)	C11B—C19B	1.422 (3)
C14A—H14A	0.9300	C12B—C13B	1.413 (3)
C15A—C16A	1.356 (4)	C13B—C14B	1.372 (3)
C15A—C20A	1.460 (3)	C13B—C26B	1.506 (4)
C15A—C28A	1.519 (4)	C14B—C20B	1.408 (3)
C16A—C17A	1.437 (4)	C14B—H14B	0.9300
C17A—C18A	1.430 (3)	C15B—C16B	1.344 (4)
C18A—C27A	1.402 (3)	C15B—C20B	1.453 (3)
C18A—C19A	1.458 (3)	C15B—C28B	1.523 (4)
C19A—C20A	1.426 (3)	C16B—C17B	1.444 (3)
C21A—H21A	0.9600	C17B—C18B	1.418 (3)
C21A—H21B	0.9600	C18B—C27B	1.410 (3)
C21A—H21C	0.9600	C18B—C19B	1.464 (3)
C22A—H22A	0.9300	C19B—C20B	1.420 (3)
C23A—C25A	1.515 (5)	C21B—H21D	0.9600
C23A—C24A	1.544 (4)	C21B—H21F	0.9600
C23A—H23A	0.9800	C21B—H21E	0.9600
C24A—H24A	0.9600	C22B—H22B	0.9300
C24A—H24B	0.9600	C23B—C24B	1.476 (6)
C24A—H24C	0.9600	C23B—C25B	1.542 (6)
C25A—H25A	0.9600	C23B—H23B	0.9800
C25A—H25B	0.9600	C24B—H24D	0.9600
C25A—H25C	0.9600	C24B—H24E	0.9600
C26A—H26A	0.9600	C24B—H24F	0.9600
C26A—H26B	0.9600	C25B—H25D	0.9600
C26A—H26C	0.9600	C25B—H25E	0.9600
C27A—H27A	0.9300	C25B—H25F	0.9600
C28A—C30A	1.434 (6)	C26B—H26F	0.9600
C28A—C30C	1.488 (9)	C26B—H26D	0.9600
C28A—C29A	1.488 (7)	C26B—H26E	0.9600
C28A—C29C	1.581 (8)	C27B—H27B	0.9300
C28A—H28A	0.9800	C28B—C30B	1.517 (4)
C28A—H28C	0.9800	C28B—C29B	1.522 (4)
C29A—H29A	0.9600	C28B—H28B	0.9800
C29A—H29B	0.9600	C29B—H29E	0.9600
C29A—H29C	0.9600	C29B—H29D	0.9600
C30A—H30A	0.9600	C29B—H29F	0.9600
C30A—H30B	0.9600	C30B—H30D	0.9600
C30A—H30C	0.9600	C30B—H30E	0.9600
C29C—H29G	0.9600	C30B—H30F	0.9600
C29C—H29H	0.9600	C31B—C32B	1.497 (4)
C29C—H29I	0.9600	C31B—H31C	0.9700
C30C—H30G	0.9600	C31B—H31D	0.9700
C30C—H30H	0.9600	C32B—C33B	1.350 (5)
C30C—H30I	0.9600	C32B—C37B	1.378 (4)
C31A—C32A	1.531 (5)	C33B—C34B	1.366 (5)
C31A—H31A	0.9700	C33B—H33B	0.9300
C31A—H31B	0.9700	C34B—C35B	1.373 (6)
C32A—C37A	1.320 (5)	C34B—H34B	0.9300
C32A—C33A	1.394 (5)	C35B—C36B	1.324 (5)
C33A—C34A	1.346 (5)	C35B—H35B	0.9300
C33A—H33A	0.9300	C36B—C37B	1.375 (4)
C34A—C35A	1.315 (6)	C36B—H36B	0.9300
C34A—H34A	0.9300	C37B—H37B	0.9300
C35A—C36A	1.389 (7)	C38B—C39B	1.492 (4)
C35A—H35A	0.9300	C38B—H38C	0.9700
C36A—C37A	1.378 (6)	C38B—H38D	0.9700
C36A—H36A	0.9300	C39B—C44B	1.341 (5)
C37A—H37A	0.9300	C39B—C40B	1.358 (5)
C38A—C39A	1.506 (4)	C40B—C41B	1.394 (7)
C38A—H38A	0.9700	C40B—H40B	0.9300
C38A—H38B	0.9700	C41B—C42B	1.335 (9)
C39A—C40A	1.343 (5)	C41B—H41B	0.9300
C39A—C44A	1.367 (5)	C42B—C43B	1.308 (8)
C40A—C41A	1.392 (6)	C42B—H42B	0.9300
C40A—H40A	0.9300	C43B—C44B	1.412 (7)
C41A—C42A	1.349 (7)	C43B—H43B	0.9300
C41A—H41A	0.9300	C44B—H44B	0.9300
C42A—C43A	1.348 (8)		
			
C1A—O1A—H1A	111 (3)	C40A—C41A—H41A	119.7
C6A—O4A—H4AB	99 (3)	C43A—C42A—C41A	118.9 (6)
C11A—O5A—H5A	113 (2)	C43A—C42A—H42A	120.5
C16A—O8A—H8A	101 (3)	C41A—C42A—H42A	120.5
C22A—N1A—C31A	127.0 (3)	C44A—C43A—C42A	120.4 (6)
C22A—N1A—H1AN	116.5	C44A—C43A—H43A	119.8
C31A—N1A—H1AN	116.5	C42A—C43A—H43A	119.8
C27A—N2A—C38A	123.4 (3)	C43A—C44A—C39A	122.5 (5)
C27A—N2A—H2AN	118.3	C43A—C44A—H44A	118.8
C38A—N2A—H2AN	118.3	C39A—C44A—H44A	118.8
O1A—C1A—C2A	119.7 (2)	C1B—O1B—H1B	107 (2)
O1A—C1A—C9A	116.5 (2)	C6B—O4B—H4BC	102 (3)
C2A—C1A—C9A	123.7 (2)	C11B—O5B—H5B	114 (2)
C1A—C2A—C3A	118.7 (2)	C16B—O8B—H8B	99 (3)
C1A—C2A—C12A	119.9 (2)	C22B—N1B—C31B	123.8 (3)
C3A—C2A—C12A	121.3 (2)	C22B—N1B—H1BN	118.1
C4A—C3A—C2A	118.6 (2)	C31B—N1B—H1BN	118.1
C4A—C3A—C21A	120.6 (2)	C27B—N2B—C38B	124.5 (2)
C2A—C3A—C21A	120.8 (2)	C27B—N2B—H2BN	117.8
C3A—C4A—C10A	123.7 (2)	C38B—N2B—H2BN	117.8
C3A—C4A—H4	118.2	O1B—C1B—C2B	119.1 (2)
C10A—C4A—H4	118.2	O1B—C1B—C9B	117.3 (2)
C6A—C5A—C10A	118.0 (2)	C2B—C1B—C9B	123.6 (2)
C6A—C5A—C23A	119.8 (2)	C1B—C2B—C3B	119.0 (2)
C10A—C5A—C23A	122.2 (2)	C1B—C2B—C12B	117.9 (2)
C5A—C6A—O4A	121.1 (3)	C3B—C2B—C12B	122.9 (2)
C5A—C6A—C7A	123.2 (2)	C4B—C3B—C2B	118.6 (2)
O4A—C6A—C7A	115.8 (2)	C4B—C3B—C21B	121.4 (2)
O3A—C7A—C8A	123.7 (2)	C2B—C3B—C21B	120.0 (2)
O3A—C7A—C6A	116.1 (2)	C3B—C4B—C10B	123.7 (3)
C8A—C7A—C6A	120.1 (2)	C3B—C4B—H4B	118.1
C7A—C8A—C22A	116.7 (2)	C10B—C4B—H4B	118.1
C7A—C8A—C9A	118.6 (2)	C6B—C5B—C10B	118.1 (3)
C22A—C8A—C9A	124.7 (2)	C6B—C5B—C23B	120.8 (3)
C10A—C9A—C1A	116.4 (2)	C10B—C5B—C23B	121.0 (3)
C10A—C9A—C8A	119.1 (2)	C5B—C6B—O4B	122.3 (3)
C1A—C9A—C8A	124.5 (2)	C5B—C6B—C7B	123.9 (2)
C4A—C10A—C9A	118.8 (2)	O4B—C6B—C7B	113.8 (3)
C4A—C10A—C5A	120.2 (2)	O3B—C7B—C8B	124.3 (3)
C9A—C10A—C5A	121.0 (2)	O3B—C7B—C6B	116.6 (2)
O5A—C11A—C12A	120.2 (2)	C8B—C7B—C6B	119.1 (3)
O5A—C11A—C19A	116.7 (2)	C22B—C8B—C7B	117.1 (2)
C12A—C11A—C19A	123.1 (2)	C22B—C8B—C9B	123.9 (2)
C11A—C12A—C13A	119.0 (2)	C7B—C8B—C9B	119.0 (2)
C11A—C12A—C2A	121.1 (2)	C1B—C9B—C10B	116.5 (2)
C13A—C12A—C2A	119.9 (2)	C1B—C9B—C8B	124.1 (2)
C14A—C13A—C12A	118.7 (2)	C10B—C9B—C8B	119.4 (2)
C14A—C13A—C26A	119.9 (2)	C4B—C10B—C9B	118.5 (2)
C12A—C13A—C26A	121.4 (2)	C4B—C10B—C5B	121.0 (3)
C13A—C14A—C20A	123.2 (2)	C9B—C10B—C5B	120.5 (2)
C13A—C14A—H14A	118.4	O5B—C11B—C12B	119.9 (2)
C20A—C14A—H14A	118.4	O5B—C11B—C19B	117.0 (2)
C16A—C15A—C20A	117.3 (2)	C12B—C11B—C19B	123.1 (2)
C16A—C15A—C28A	120.3 (2)	C11B—C12B—C13B	119.3 (2)
C20A—C15A—C28A	122.4 (2)	C11B—C12B—C2B	120.8 (2)
C15A—C16A—O8A	121.6 (3)	C13B—C12B—C2B	119.9 (2)
C15A—C16A—C17A	124.7 (2)	C14B—C13B—C12B	118.3 (2)
O8A—C16A—C17A	113.6 (2)	C14B—C13B—C26B	121.0 (2)
O7A—C17A—C18A	123.8 (2)	C12B—C13B—C26B	120.7 (2)
O7A—C17A—C16A	118.2 (2)	C13B—C14B—C20B	123.5 (2)
C18A—C17A—C16A	118.0 (2)	C13B—C14B—H14B	118.2
C27A—C18A—C17A	117.4 (2)	C20B—C14B—H14B	118.2
C27A—C18A—C19A	123.6 (2)	C16B—C15B—C20B	118.3 (2)
C17A—C18A—C19A	118.7 (2)	C16B—C15B—C28B	122.9 (2)
C11A—C19A—C20A	117.1 (2)	C20B—C15B—C28B	118.8 (2)
C11A—C19A—C18A	123.5 (2)	C15B—C16B—O8B	122.9 (2)
C20A—C19A—C18A	119.4 (2)	C15B—C16B—C17B	123.8 (2)
C14A—C20A—C19A	118.7 (2)	O8B—C16B—C17B	113.3 (2)
C14A—C20A—C15A	120.8 (2)	O7B—C17B—C18B	123.5 (2)
C19A—C20A—C15A	120.5 (2)	O7B—C17B—C16B	117.2 (2)
C3A—C21A—H21A	109.5	C18B—C17B—C16B	119.3 (2)
C3A—C21A—H21B	109.5	C27B—C18B—C17B	117.5 (2)
H21A—C21A—H21B	109.5	C27B—C18B—C19B	124.4 (2)
C3A—C21A—H21C	109.5	C17B—C18B—C19B	118.0 (2)
H21A—C21A—H21C	109.5	C20B—C19B—C11B	116.6 (2)
H21B—C21A—H21C	109.5	C20B—C19B—C18B	119.8 (2)
N1A—C22A—C8A	123.4 (3)	C11B—C19B—C18B	123.6 (2)
N1A—C22A—H22A	118.3	C14B—C20B—C19B	119.0 (2)
C8A—C22A—H22A	118.3	C14B—C20B—C15B	120.5 (2)
C5A—C23A—C25A	110.5 (3)	C19B—C20B—C15B	120.6 (2)
C5A—C23A—C24A	114.7 (3)	C3B—C21B—H21D	109.5
C25A—C23A—C24A	111.0 (3)	C3B—C21B—H21F	109.5
C5A—C23A—H23A	106.7	H21D—C21B—H21F	109.5
C25A—C23A—H23A	106.7	C3B—C21B—H21E	109.5
C24A—C23A—H23A	106.7	H21D—C21B—H21E	109.5
C23A—C24A—H24A	109.5	H21F—C21B—H21E	109.5
C23A—C24A—H24B	109.5	N1B—C22B—C8B	124.3 (3)
H24A—C24A—H24B	109.5	N1B—C22B—H22B	117.8
C23A—C24A—H24C	109.5	C8B—C22B—H22B	117.8
H24A—C24A—H24C	109.5	C24B—C23B—C5B	111.1 (4)
H24B—C24A—H24C	109.5	C24B—C23B—C25B	113.9 (4)
C23A—C25A—H25A	109.5	C5B—C23B—C25B	114.2 (4)
C23A—C25A—H25B	109.5	C24B—C23B—H23B	105.6
H25A—C25A—H25B	109.5	C5B—C23B—H23B	105.6
C23A—C25A—H25C	109.5	C25B—C23B—H23B	105.6
H25A—C25A—H25C	109.5	C23B—C24B—H24D	109.5
H25B—C25A—H25C	109.5	C23B—C24B—H24E	109.5
C13A—C26A—H26A	109.5	H24D—C24B—H24E	109.5
C13A—C26A—H26B	109.5	C23B—C24B—H24F	109.5
H26A—C26A—H26B	109.5	H24D—C24B—H24F	109.5
C13A—C26A—H26C	109.5	H24E—C24B—H24F	109.5
H26A—C26A—H26C	109.5	C23B—C25B—H25D	109.5
H26B—C26A—H26C	109.5	C23B—C25B—H25E	109.5
N2A—C27A—C18A	125.4 (2)	H25D—C25B—H25E	109.5
N2A—C27A—H27A	117.3	C23B—C25B—H25F	109.5
C18A—C27A—H27A	117.3	H25D—C25B—H25F	109.5
C30A—C28A—C30C	109.3 (12)	H25E—C25B—H25F	109.5
C30A—C28A—C29A	115.9 (5)	C13B—C26B—H26F	109.5
C30C—C28A—C29A	15.2 (12)	C13B—C26B—H26D	109.5
C30A—C28A—C15A	119.1 (3)	H26F—C26B—H26D	109.5
C30C—C28A—C15A	124.4 (10)	C13B—C26B—H26E	109.5
C29A—C28A—C15A	111.1 (4)	H26F—C26B—H26E	109.5
C30A—C28A—C29C	51.0 (5)	H26D—C26B—H26E	109.5
C30C—C28A—C29C	118.6 (10)	N2B—C27B—C18B	125.2 (2)
C29A—C28A—C29C	133.7 (6)	N2B—C27B—H27B	117.4
C15A—C28A—C29C	112.7 (5)	C18B—C27B—H27B	117.4
C30A—C28A—H28A	102.6	C30B—C28B—C29B	111.0 (2)
C30C—C28A—H28A	91.4	C30B—C28B—C15B	117.6 (2)
C29A—C28A—H28A	102.6	C29B—C28B—C15B	108.9 (2)
C15A—C28A—H28A	102.6	C30B—C28B—H28B	106.2
C29C—C28A—H28A	53.6	C29B—C28B—H28B	106.2
C30A—C28A—H28C	46.8	C15B—C28B—H28B	106.2
C30C—C28A—H28C	97.0	C28B—C29B—H29E	109.5
C29A—C28A—H28C	92.1	C28B—C29B—H29D	109.5
C15A—C28A—H28C	97.0	H29E—C29B—H29D	109.5
C29C—C28A—H28C	97.0	C28B—C29B—H29F	109.5
H28A—C28A—H28C	149.2	H29E—C29B—H29F	109.5
C28A—C29A—H29A	109.5	H29D—C29B—H29F	109.5
C28A—C29A—H29B	109.5	C28B—C30B—H30D	109.5
C28A—C29A—H29C	109.5	C28B—C30B—H30E	109.5
C28A—C30A—H30A	109.5	H30D—C30B—H30E	109.5
C28A—C30A—H30B	109.5	C28B—C30B—H30F	109.5
C28A—C30A—H30C	109.5	H30D—C30B—H30F	109.5
C28A—C29C—H29G	109.5	H30E—C30B—H30F	109.5
C28A—C29C—H29H	109.5	N1B—C31B—C32B	114.3 (3)
H29G—C29C—H29H	109.5	N1B—C31B—H31C	108.7
C28A—C29C—H29I	109.5	C32B—C31B—H31C	108.7
H29G—C29C—H29I	109.5	N1B—C31B—H31D	108.7
H29H—C29C—H29I	109.5	C32B—C31B—H31D	108.7
C28A—C30C—H30G	109.5	H31C—C31B—H31D	107.6
C28A—C30C—H30H	109.5	C33B—C32B—C37B	118.0 (3)
H30G—C30C—H30H	109.5	C33B—C32B—C31B	118.0 (3)
C28A—C30C—H30I	109.5	C37B—C32B—C31B	123.9 (3)
H30G—C30C—H30I	109.5	C32B—C33B—C34B	121.6 (4)
H30H—C30C—H30I	109.5	C32B—C33B—H33B	119.2
N1A—C31A—C32A	115.0 (3)	C34B—C33B—H33B	119.2
N1A—C31A—H31A	108.5	C33B—C34B—C35B	119.7 (4)
C32A—C31A—H31A	108.5	C33B—C34B—H34B	120.2
N1A—C31A—H31B	108.5	C35B—C34B—H34B	120.2
C32A—C31A—H31B	108.5	C36B—C35B—C34B	119.2 (4)
H31A—C31A—H31B	107.5	C36B—C35B—H35B	120.4
C37A—C32A—C33A	117.5 (4)	C34B—C35B—H35B	120.4
C37A—C32A—C31A	118.8 (4)	C35B—C36B—C37B	121.6 (4)
C33A—C32A—C31A	123.6 (3)	C35B—C36B—H36B	119.2
C34A—C33A—C32A	118.7 (4)	C37B—C36B—H36B	119.2
C34A—C33A—H33A	120.7	C36B—C37B—C32B	119.8 (4)
C32A—C33A—H33A	120.7	C36B—C37B—H37B	120.1
C35A—C34A—C33A	124.9 (5)	C32B—C37B—H37B	120.1
C35A—C34A—H34A	117.5	N2B—C38B—C39B	113.5 (2)
C33A—C34A—H34A	117.5	N2B—C38B—H38C	108.9
C34A—C35A—C36A	116.8 (5)	C39B—C38B—H38C	108.9
C34A—C35A—H35A	121.6	N2B—C38B—H38D	108.9
C36A—C35A—H35A	121.6	C39B—C38B—H38D	108.9
C37A—C36A—C35A	119.0 (5)	H38C—C38B—H38D	107.7
C37A—C36A—H36A	120.5	C44B—C39B—C40B	118.3 (4)
C35A—C36A—H36A	120.5	C44B—C39B—C38B	121.6 (4)
C32A—C37A—C36A	123.0 (5)	C40B—C39B—C38B	120.0 (3)
C32A—C37A—H37A	118.5	C39B—C40B—C41B	120.6 (5)
C36A—C37A—H37A	118.5	C39B—C40B—H40B	119.7
N2A—C38A—C39A	113.7 (3)	C41B—C40B—H40B	119.7
N2A—C38A—H38A	108.8	C42B—C41B—C40B	119.9 (7)
C39A—C38A—H38A	108.8	C42B—C41B—H41B	120.0
N2A—C38A—H38B	108.8	C40B—C41B—H41B	120.0
C39A—C38A—H38B	108.8	C43B—C42B—C41B	120.4 (7)
H38A—C38A—H38B	107.7	C43B—C42B—H42B	119.8
C40A—C39A—C44A	117.8 (4)	C41B—C42B—H42B	119.8
C40A—C39A—C38A	122.4 (3)	C42B—C43B—C44B	120.6 (6)
C44A—C39A—C38A	119.8 (3)	C42B—C43B—H43B	119.7
C39A—C40A—C41A	119.8 (5)	C44B—C43B—H43B	119.7
C39A—C40A—H40A	120.1	C39B—C44B—C43B	120.1 (5)
C41A—C40A—H40A	120.1	C39B—C44B—H44B	119.9
C42A—C41A—C40A	120.6 (6)	C43B—C44B—H44B	119.9
C42A—C41A—H41A	119.7		

### Hydrogen-bond geometry (Å, º)

**Table:**

*D*—H···*A*	*D*—H	H···*A*	*D*···*A*	*D*—H···*A*
N1*A*—H1*AN*···O3*A*	0.86	1.79	2.494 (3)	138
N1*B*—H1*BN*···O3*B*	0.86	1.85	2.531 (3)	135
N2*A*—H2*AN*···O7*A*	0.86	1.93	2.584 (3)	132
N2*B*—H2*BN*···O7*B*	0.86	1.89	2.561 (3)	134
O1*A*—H1*A*···O7*B*^i^	0.65 (2)	2.24 (3)	2.778 (3)	141 (3)
O1*B*—H1*B*···O7*A*^ii^	0.77 (3)	2.33 (3)	2.846 (2)	125 (3)
O4*A*—H4*AB*···O3*A*	0.74 (3)	2.05 (3)	2.588 (3)	130 (3)
O4*B*—H4*BC*···O3*B*	0.83 (4)	1.98 (4)	2.568 (4)	126 (4)
O5*A*—H5*A*···O3*B*	0.76 (3)	2.10 (3)	2.714 (3)	138 (3)
O5*B*—H5*B*···O3*A*	0.81 (3)	1.98 (3)	2.645 (3)	139 (3)
O8*A*—H8*A*···O1*B*^iii^	0.92 (5)	2.45 (5)	3.290 (3)	151 (4)
O8*A*—H8*A*···O7*A*	0.92 (5)	1.95 (5)	2.592 (3)	126 (4)
O8*B*—H8*B*···O1*A*^iv^	0.86 (5)	2.40 (5)	3.183 (3)	151 (4)
O8*B*—H8*B*···O7*B*	0.86 (5)	1.93 (5)	2.577 (3)	130 (4)

Symmetry codes: (i) −*x*−1, *y*+1/2, −*z*+1/2; (ii) −*x*, *y*−1/2, −*z*+1/2; (iii) −*x*, *y*+1/2, −*z*+1/2; (iv) −*x*−1, *y*−1/2, −*z*+1/2.
